# Influence of Mullite and Halloysite Reinforcement on the Ablation Properties of an Epoxy Composite

**DOI:** 10.3390/ma18153530

**Published:** 2025-07-28

**Authors:** Robert Szczepaniak, Michał Piątkiewicz, Dominik Gryc, Paweł Przybyłek, Grzegorz Woroniak, Joanna Piotrowska-Woroniak

**Affiliations:** 1 Faculty of Aviation, Polish Air Force University, Dywizjonu 303 No. 35, 08-521 Deblin, Poland; p.przybylek@law.mil.pl; 2 41 Air Training Base, Brygady Poscigowej 5, 08-521 Deblin, Poland; michal.piatkiewicz3@gmail.com; 3 Institute of Navigation, Polish Air Force University, Dywizjonu 303 No. 35, 08-521 Deblin, Poland; d.gryc3615@gmail.com; 4 HVAC Department, Bialystok University of Technology, Wiejska 45E, 15-351 Bialystok, Poland; g.woroniak@pb.edu.pl (G.W.); j.piotrowska@pb.edu.pl (J.P.-W.)

**Keywords:** ablative properties, halloysite, mullite, ablation, thermal protection

## Abstract

This paper explores the impact of applying a powder additive in the form of halloysite and mullite on the thermal protection properties of a composite. The authors used CES R70 epoxy resin with CES H72 hardener, modified by varying the amount of powder additive. The composite samples were exposed to a mixture of combustible gases at a temperature of approximately 1000 °C. The primary parameters analyzed during this study were the temperature on the rear surface of the sample and the ablative mass loss of the tested material. The temperature increase on the rear surface of the sample, which was exposed to the hot stream of flammable gases, was measured for 120 s. Another key parameter considered in the data analysis was the ablative mass loss. The charred layer of the sample played a crucial role in this process, as it helped block oxygen diffusion from the boundary layer of the original material. This charred layer absorbed thermal energy until it reached a temperature at which it either oxidized or was mechanically removed due to the erosive effects of the heating factor. The incorporation of mullite reduced the rear surface temperature from 58.9 °C to 49.2 °C, and for halloysite, it was reduced the rear surface temperature to 49.8 °C. The ablative weight loss dropped from 57% to 18.9% for mullite and to 39.9% for halloysite. The speed of mass ablation was reduced from 77.9 mg/s to 25.2 mg/s (mullite) and 52.4 mg/s (halloysite), while the layer thickness loss decreased from 7.4 mm to 2.8 mm (mullite) and 4.4 mm (halloysite). This research is innovative in its use of halloysite and mullite as functional additives to enhance the ablative resistance of polymer composites under extreme thermal conditions. This novel approach not only contributes to a deeper understanding of composite behavior at high temperatures but also opens up new avenues for the development of advanced thermal protection systems. Potential applications of these materials include aerospace structures, fire-resistant components, and protective coatings in environments exposed to intense heat and flame.

## 1. Introduction

Thermal insulation for sensitive aerospace components like flight data recorders, aerodynamic surfaces, missile loads, and space shuttles is crucial for safety and efficiency during hypersonic flights. High speeds cause a significant temperature rise in heated elements. Ablative materials can create thermal barriers to manage this heat flow. Research into various ablative materials and additives has been ongoing since humans achieved high speeds in their constructions.

Currently, there is a focus on enhancing both the ablation and thermal characteristics of materials as well as their mechanical properties. Research is being conducted to understand the impact of mechanical loads and deformations on composite materials when analyzing phenomena related to the influence of heat flux [1]. Studies have examined the effects of the arrangement of individual layers in laminates and the criteria for composite failure; for instance, Griffis [2]. Dodds [3] considered additional endothermic transformations in the matrix material and the convective effect of gases generated during ablation. The results of these studies aligned with experimental findings concerning heat transfer effects but did not account for the influence of mechanical loads. Other researchers, such as Komorek et al. [4], Kucharczyk et al. [5], Wagih et al. [6], and Guerrero et al. [7], focused on similar investigations into the thermomechanical properties of epoxy composites. Materials like C/C, C/SiC, and SiC/SiC composites are preferred due to their optimal strength properties and high heat resistance [8,9,10,11]. C/SiC composites, in particular, possess good thermal resistance, adequate strength properties, oxidation resistance, and decreasing production costs [12,13,14]. Polymer coatings with ablative composite matrices, such as epoxy resins [1,2,3,15,16] or phenolic resins [17,18,19,20,21,22,23,24], along with fillers to enhance thermal stability [1,2,12,15,16,17,18,21,25,26,27], can provide improved thermal protection. However, ablative resins require reinforcement due to their porosity, low decomposition temperature, and brittleness. Research has explored reinforcement with fibers [8,9,10,11,16,17,18,28,29,30,31], powder additions [1,15,21,32,33,34], and fillings such as boards and aerogels [33,35,36,37,38,39]. Alagar et al. [40] proposed the use of Kevlar 49, and Kucharczyk et al. [41] tested aramid fibers in thermo-protective ablative castings, demonstrating that the introduction of additives can raise the melting point and enhance the thermomechanical and thermo-protective properties of the ablative composite.

Ablation is a heat and mass transfer process that leads to irreversible structural and chemical changes in a material due to physical changes and chemical reactions accompanied by heat absorption. This process is driven by external sources of thermal energy [42,43,44]. There are several physical types of ablation, including superficial wall ablation (without aerodynamic erosion on the ablation surface), wall ablation with secondary ablation processes in the ablation layer, superficial wall ablation with aerodynamic erosion on the ablation surface, and wall ablation with aerodynamic erosion on the ablation surface with secondary ablation in the ablation layer [45]. No universal model describes the processes occurring in the composite material under the influence of heat flux. It is extremely difficult to provide a detailed description of the chemical reactions and physical phenomena during combustion in the composite material [46].

**Figure 1:**
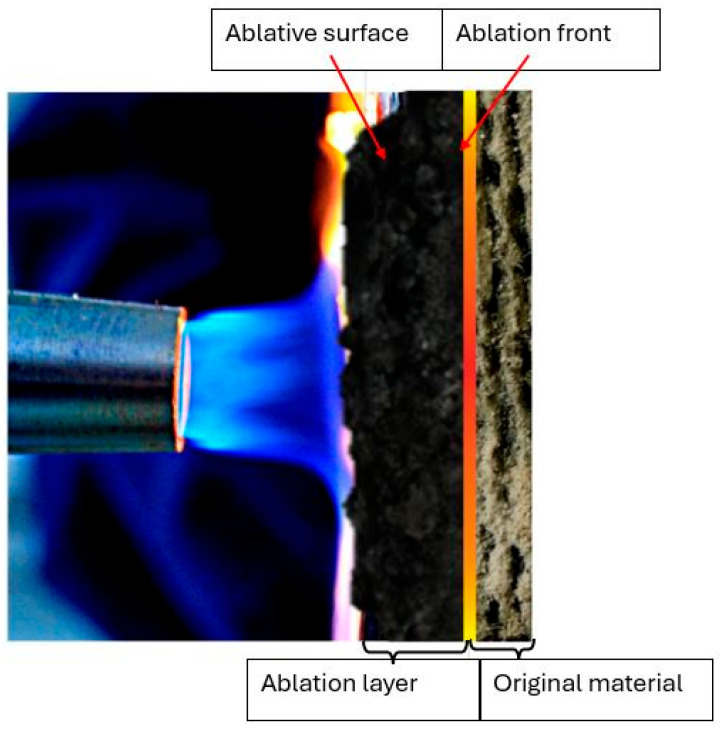
Diagram of the physical model of ablation.

During the ablation process in materials, distinct layers can be observed (see Figure 1) at the interface between the gas and solid phases, forming the ablation surface. The boundary surface that separates the ablative layer from the original material is termed the ablative front [47].

Properly utilizing the material’s ablative properties, i.e., reducing the active volume of the material not subject to ablation, requires consideration and understanding of the material’s ablative wear mechanism.

Based on [48], the adequate ablation heat of the material *H* is expressed by the relationship between the heat flux density and the mass ablation rate.

(1) $$H=\frac{q_{0}}{\dot{m}}=\{\int_{T_{o}}^{T_{a}}c_{p}dT+\xi \left[c_{f}+\beta {\left(∆H\right)}_{0}\right]\}\cdot \left(\frac{q_{0}}{q_{o}-q_{p}}\right)$$

where:

$\dot{m}$—massive ablation rate [g/s],
$q_{0}$—heat flux density determined for the original (not degraded) surface at the ablation temperature [W/m^2^],
$q_{p}$—emission flux density [W/m^2^],
$c_{f}$—the heat of phase changes (melting, evaporation, sublimation) [J/kg],
$c_{p}$—specific heat at constant pressure [J/kgK],
$T_{a}$—absolute ablation temperature on the material surface [K],
∆H—gas enthalpy increase in the boundary layer [J/kg],
ξ—gasification coefficient of pyrolysis products,
β—mass exchange coefficient with air.

The thermal diffusivity of a material determines its ability to equalize temperature gradients. Therefore, developing ablative thermal protection involves finding materials with high density (*ρ*) and high specific heat (*C_p_*(*t*)) while also having a low thermal conductivity coefficient (*λ*(*t*)); in other words, low thermal diffusivity (as referenced in [42]).

(2) $$\alpha \left(t\right)=\frac{\lambda (t)}{{\rho C}_{p}(t)}$$

Mullite, with a high melting point of 1850 °C, is the sole stable compound in the Al_2_O_3_–SiO_2_ binary system [49,50]. High-purity mullite ceramic materials serve as essential raw materials in the production of functional ceramics and find extensive applications in metallurgy, energy conservation, environmental protection, and the electronics industry. This is attributed to their remarkably high-temperature strength properties, low thermal expansion coefficient, and excellent chemical stability [51,52,53,54]. The exceptional high-temperature properties of mullite render it an appealing choice for deployment in various applications, including as a matrix material for the production of high-temperature composites, a substrate in multilayer packaging and protective coatings, and an infrared transparent window specifically tailored for high-temperature applications [55,56,57,58]. Mullite is a preferred ceramic material for thermal protection systems (TPSs) due to its excellent thermal resistance, oxidation resistance, low thermal expansion coefficient, mechanical strength, high creep resistance at low and high temperatures, as well as good chemical and thermal stability [59]. Mullite matrix composites are used in various components and constructions, such as gas turbine engines (inserts, thermal shingles for combustion chambers, exhaust cones), high-efficiency furnaces, burner tubes, and heat shields for space vehicles during entry [60,61]. The addition of reinforced fibers significantly enhances the mechanical properties of the mullite and could promote electromagnetic wave absorption [62,63]. From the literature preview, it appears that using mullite in epoxy composites is uncommon.

Halloysite (Hal) is a material that has been studied for its impact on the ablative properties of epoxy composites [64,65]. It is a natural clay with a tubular structure suitable for creating hybrids/composites. Halloysite has gained attention due to its environmentally friendly nature and availability in large quantities for industrial use. Its unique chemical properties allow for the formation of new structures with other aluminosilicates, such as zeolites, while retaining its functional properties [66]. Halloysite nanotubes (HNTs) are naturally occurring aluminosilicates with a hollow tubular structure mined from natural deposits [67,68,69,70,71]. These nanotubes can be used to reinforce polymers and have a unique strengthening effect on various polymers [72,73,74,75,76,77]. Halloysite nanotubes (HNTs) allow for smooth diffusion in the polymer matrix compared to other nano-clays [78,79,80]. This makes HNTs an excellent candidate for polymer composites and has sparked significant research interest [77]. As an additive, HNTs are used to improve mechanical and/or thermal properties [81,82].

Utilizing well-dispersed nanofillers such as halloysite nanotubes in nanocomposites has enhanced fire resistance and thermal stability [83]. Halloysite nanotubes (HNTs) offer a promising alternative as flame-retardant carriers for controlling the migration of flame-retardant molecules, thereby providing long-term aging properties. A review by Jasinski et al. [66] highlighted the impact of HNTs and their derivatives on the fire-resistant properties of polymer-based composites, emphasizing the superior flame-retardant properties of halloysite nanotubes. HNTs can serve as flame retardants by forming a ceramic structure and creating a barrier against heat and mass transfer.

The mechanical properties of exfoliated clay/epoxy nanocomposites have attracted significant attention due to the properties and expanding range of applications. Numerous researchers have conducted numerical studies in this area [84,85,86,87,88,89,90,91]. For instance, Zhao et al. [88] studied the impact of micromechanics models on the prediction of the mechanical properties of short fiber composites. Khudari Bek et al. [89] presented a new mesh algorithm for investigating the elastic properties of nanocomposite polymeric materials (PNCs) using the scaled boundary finite element method (SBFEM). Shi et al. [91] conducted a study on a structurally integrated thermal protection system (ITPS) with both load capacity and thermal protection. They tested an innovative, fully composite sandwich panel with a corrugated core manufactured by hot pressing. They presented an analytical model of the composite sandwich panel based on the theory of surface ablation and transient heat transfer, which considers pyrolysis reactions, phase transitions, evaporation of quartz fibers, formation of decomposition gases, and surface ablation.

Considering the above, the authors decided to investigate the ablation properties of the epoxy composite after adding mullite and halloysite, with the aim of developing advanced thermal protection materials. This study focused on evaluating the impact of these ceramic additives on key performance indicators such as the rear surface temperature, ablative mass loss, and material thickness reduction under extreme thermal loads. The innovative aspect of this research lies in the use of mullite and halloysite as functional fillers in an epoxy matrix, which has not been widely explored in the context of high-temperature ablative applications. By comparing the effects of both additives, this study also aimed to determine their relative effectiveness and potential for optimizing composite formulations intended for aerospace, defense, or fire-resistant technologies.

## 2. Materials and Methods

Ablative materials have the ability to dissipate significant amounts of thermal energy through phase changes, making them ideal for protecting various components from the effects of high-temperature heat flux.

Due to the growing demand for materials resistant to degradation during prolonged exposure to elevated temperatures [26,92], an investigation was conducted to assess the impact of adding mullite and halloysite on the thermal resistance of a polymer composite. To determine the ablation properties of the tested sample structure, the following aspects were analyzed during the research process:

(1) temperature of the back surface of the tested material Ts,
(2) temperature of the ablation surface Ta,
(3) relative ablative loss of mass M_a_,
(4) relative speed of mass ablation m_a_,
(5) geometric defect of the ablation layer l.

### 2.1. Material Subjected to Ablation Tests

For the tests, a composite was made based on the CES R70 epoxy resin [50] cross-linked at room temperature using the CES H72 hardener [49] manufactured by SIKA. This resin is widely used, among others, for gluing, laminating, casting, and flooring systems. The density of the resin is 1.16 ± 0.02 g/cm^3^, and the density of the hardener is 1.02 ± 0.02 g/cm^3^. The working time of the resin and hardener mixture is approximately 45 min at room temperature. The manufacturer-recommended mixing ratio for the ingredients is 100:54 (CES R70H72). The filler used was mullite powder (aluminum silicate, 3Al_2_O_3_·2SiO_2_) (size < 45 µm) from Sigma Aldrich, along with halloysite nanopowder (Al_2_Si_2_O_5_(OH)_4_·2H_2_O) (diameter: 30–70 °nm × length 1–3 °μm, nanotube), also from Sigma Aldrich. The melting point of mullite is 1810 °C, with a relative density of 3.03 g/cm^3^. Halloysite, on the other hand, has a melting point above 1300 °C and a density of 2.53 g/cm^3^. Mullite (porcellanite) is a constituent mineral in thermally metamorphosed rock known as porcellanite [52]. Halloysite is a natural nanoclay with the same chemical composition as the more common kaolinite clay. Nine series of samples were prepared, each consisting of three pieces, differing in the amount of powder additive used (see Table 1). To ensure proper dispersion of the ingredients, due to the significant differences in the densities of the individual materials, the powders were added a few minutes before the resin reached its gelation point. After the powder was added, manual mixing was performed, followed by the application of ultrasonic treatment to ensure homogeneous dispersion of the additives and effective degassing.

Then, the material was gravity-cast into silicone molds with a diameter of about 38 mm and a thickness of about 12 mm. After a 7-day hardening period at room temperature, they were embedded in a plasterboard to isolate the back surface of the tested sample from the heat of the flame. For this purpose, a high-temperature sealant Ceresit CS 38 from Ceresit Co. Henkel Poland, Bydgoszcz, Poland was used, up to 1500 °C. After applying the sealant, we waited about 12 h for it to harden.

**Figure 2:**
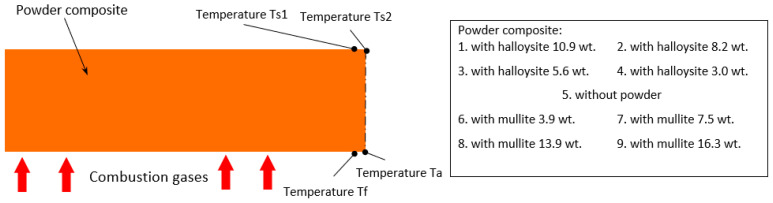
The shape of the sample subjected to ablation testing with the locations of the measurement sites for individual temperatures (9 types of composites).

During their production, thermocouples were placed in each sample to measure the temperature during the tests. The thermocouples were mounted on the back surface of the sample (temperature measurements Ts1 and Ts2) and one control element was attached to check the rate of the temperature increase on the front surface Tf (see Figure 2), which recorded the temperature only for the first seconds of measurement due to the rapid burning due to the direct impact of the flammable gas stream. The temperature of the ablation surface was also measured using an optical pyrometer Ta.

### 2.2. Test Stand and Research Methodology

**Figure 3:**
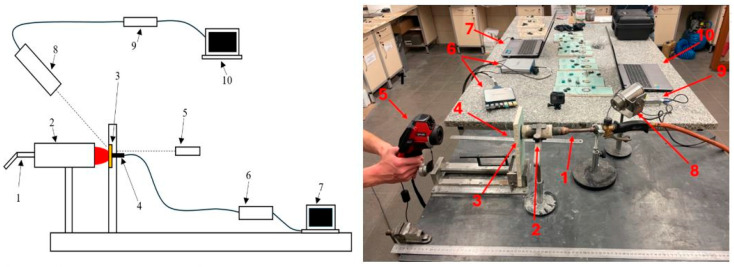
Diagram and photo of the measurement setup: 1—butane burner, 2—ablation gun with stand, 3—fireproof gypsum board and test sample, 4—thermocouples, 5—thermal imaging camera, 6—interface for reading data from thermocouples, 7—computer for recording data from thermocouples, 8—pyrometer, 9—interface for reading data from the pyrometer, 10—computer for recording data from the pyrometer.

Due to the temperature range, it was decided to measure with type J thermocouples on the rear surface of the composite and the type K on the surface on the side of the heat source (for control purposes). At the authors’ test stand (see Figure 3), samples produced using the previously described method were exposed to a stream of hot gases (approximately 1000–1100 °C). The test duration was limited to approximately 120 s for each sample.

**Figure 4:**
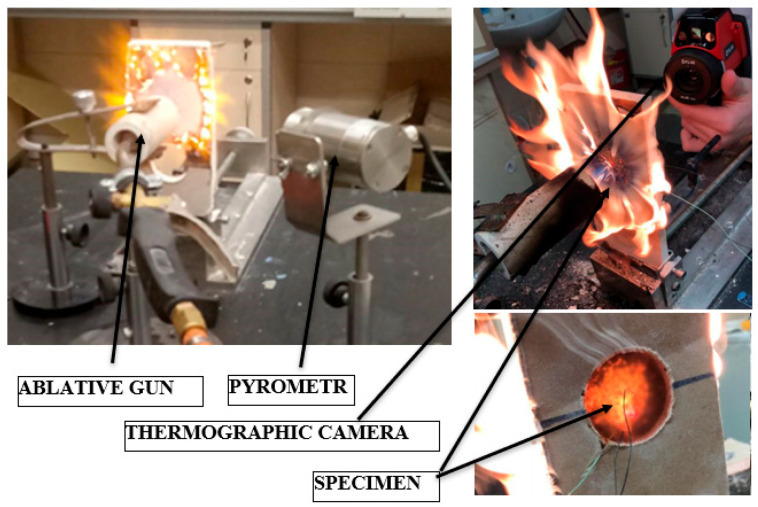
The test stand during the investigations.

To achieve a directed and focused flame, the burner was placed inside the "ablation gun," which consists of a ceramic tube (see Figure 4). The temperature was recorded using a National Instruments device, model SCB, Austin, TX, USA, in conjunction with National Instruments LabView 2015 software, Austin, TX, USA. The temperature of the ablation surface, which was exposed to the flame of the tested sample, was measured using an Optris pyrometer, model CT, Berlin, Germany, equipped with a laser pointer for precise targeting of the measurement site (see Figure 4). To ensure uniformity in the temperature distribution on the back surface of the sample, an FLIR SYSTEMS AB, FLIR i60, thermal imaging camera Arlington, VA, USA was utilized. Prior to conducting the tests, the measuring system was calibrated to ensure accuracy. Additionally, the optimum distance from the ablation gun to the ablation surface was determined to achieve a target temperature of approximately 1000 °C.

## 3. Results and Discussion

### 3.1. Measurement of the Temperature of the Ablation Surface and the Temperature on the Back Surface of the Composite

**Figure 5:**
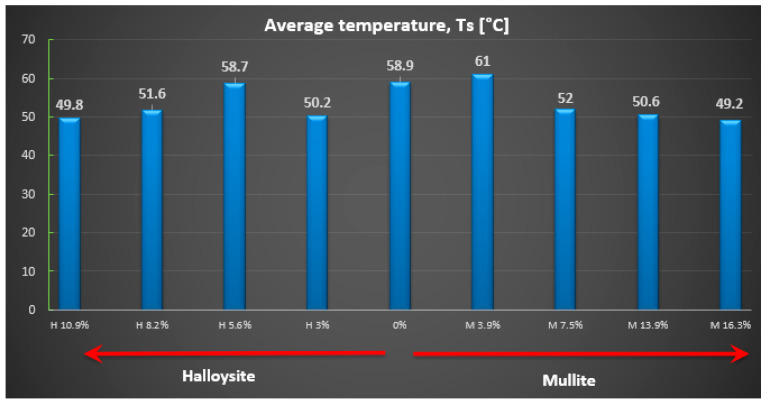
Average values of the temperature of the back surface of the wall (Ts) for individual composite series.

The most crucial parameter for analyzing the effect of powder additives on the ablative thermal protective properties of the powder-reinforced polymer composite is the change in temperature on the back surface of the tested material (denoted as Ts). To compare the influence of these additives, the authors recorded the temperature at the last second of the Ts test, specifically after 120 s of heat flow exposure to the tested material. The average temperature values for the back surface are presented (see Figure 5). 

Based on the obtained measurement results, it can be seen that when adding a powder additive in the form of halloysite and mullite, only when it reaches approximately 8% is a drop in the rear surface temperature visible (see Figure 5). The best thermal protection properties, and therefore the lowest temperature value for the back wall of the sample, are seen for composites with an increased amount of the additive. Their average temperature for the back surface of the wall was ~50 °C.

**Figure 6:**
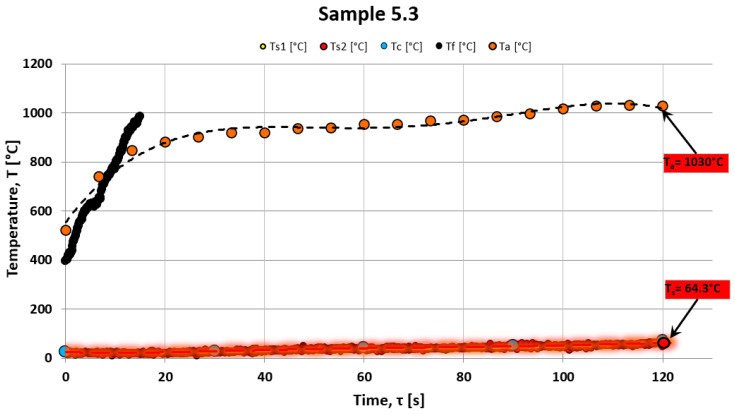
Example of the temperature increase on the back wall of the sample Ts, Tc, and the ablation surface as well as on the ablation front Tf and Ta.

Figure 6 shows an example of the temperature change over time recorded for sample 5.3, which consisted of unmodified epoxy resin without powder addition (see Figure 6). The temperature was measured on the rear surface of the sample during exposure to a high-temperature gas stream for 120 s. This temperature profile serves as a reference for evaluating the thermal insulation performance of composites modified with ceramic fillers. The relatively rapid and continuous increase in the rear surface temperature reflects the limited thermal protection capability of the base material in the absence of additives. In addition to the rear surface temperature, the results of the non-contact surface temperature measurements of the ablation zone performed using a pyrometer, as well as the position of the ablation front over time, are also presented. These measurements provide a more comprehensive view of the thermal response and material degradation mechanisms under high-temperature exposure.

**Figure 7:**
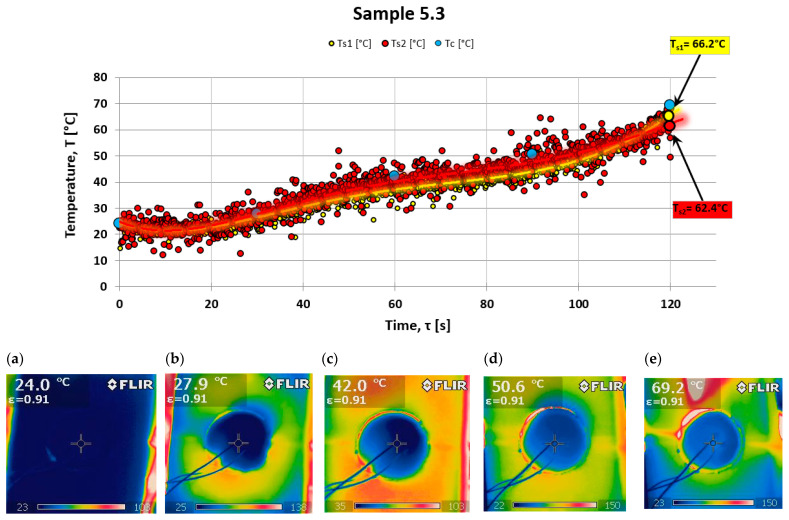
Temperature behind fiber composite Ts (thermocouple) and Tc (thermography) and a set of photos taken for series 5.3 in (**a**)—0 s; (**b**)—30 s; (**c**)—60 s; (**d**)—90 s; and (**e**)—120 s of the test.

**Figure 8:**
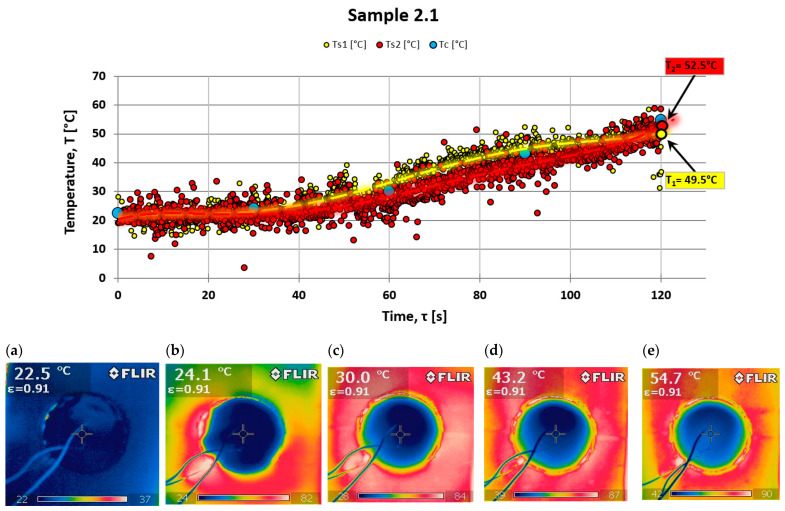
Temperature behind fiber composite Ts (thermocouple) and Tc (thermography) and a set of photos taken for series 5.3 in (**a**)—0 s; (**b**)—30 s; (**c**)—60 s; (**d**)—90 s; and (**e**)—120 s of the test.

For control purposes, an FLIR i60 thermal imaging camera was used to check the uniformity of the temperature field distribution on the back surface while testing the ablation properties of the composites. This camera allows for detailed imaging of the temperature variations. Figure 7 illustrates an example of the temperature distribution on the back surface of the tested material, specifically sample 5.3 (a composite without powder additives). Temperature readings from a point close to the center of the sample, where thermocouples were also placed, were taken every 30 s. These measured values were then plotted on a temperature distribution graph (see Figure 7 and Figure 8).

There is a significant similarity between the results obtained via these two research techniques. Two measurements from different samples are presented: one from a sample made solely of resin with a hardener and no powder addition (see Figure 7), and the other from a sample with halloysite added (see Figure 8), which demonstrates a different nature of temperature changes on the back surface.

Analyzing the results of testing the temperature increase on the rear surface for the composite with powder additive, smaller values can be noticed compared to the composite without the additive. Observing the photos obtained from a thermal imaging camera, one can notice more significant thermal blockage in the material with the addition of powder (see Figure 8). 

### 3.2. Changes in Mass and Geometric Properties of Samples During Ablation Tests

**Figure 9:**
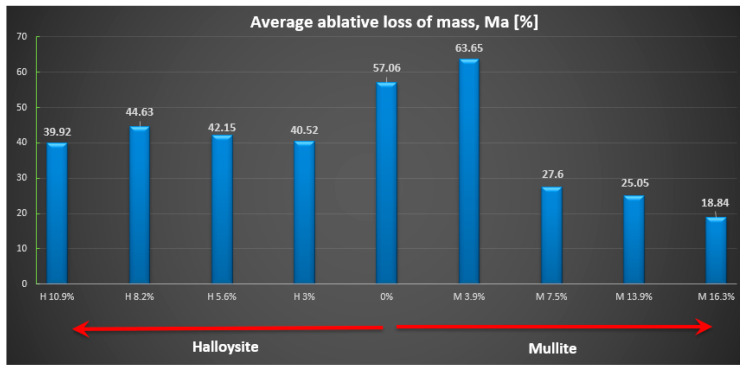
Average relative ablative loss of mass M_a_ [%].

**Figure 10:**
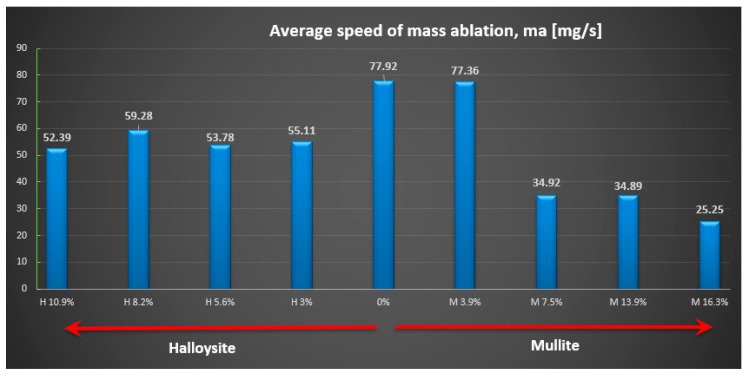
Average relative speed of mass ablation m_a_ [mg/s].

The relative ablative loss of mass (expressed as a percentage) was determined by analyzing the changes in the weight of the examined samples before and after the heat resistance tests. The obtained results are presented (see Figure 9), while the relative speed of mass loss is shown in mg/s (see Figure 10). Individual data for each sample are detailed (Table 2).

The highest ablation mass loss was recorded for the composite material without powder addition (57%) or with a small amount, especially mullite (64%), which corresponds to a mass ablation rate of 78 mg/s and 77 mg/s mass loss during the tests, so the changes were not recorded compared to the composite without the addition. In the case of the addition of halloysite, regardless of the amount, the ablation mass loss was about 40% and the ablation rate was about 56 mg/s, which is a reduction of about 30% compared to the composite without the addition.

**Figure 11:**
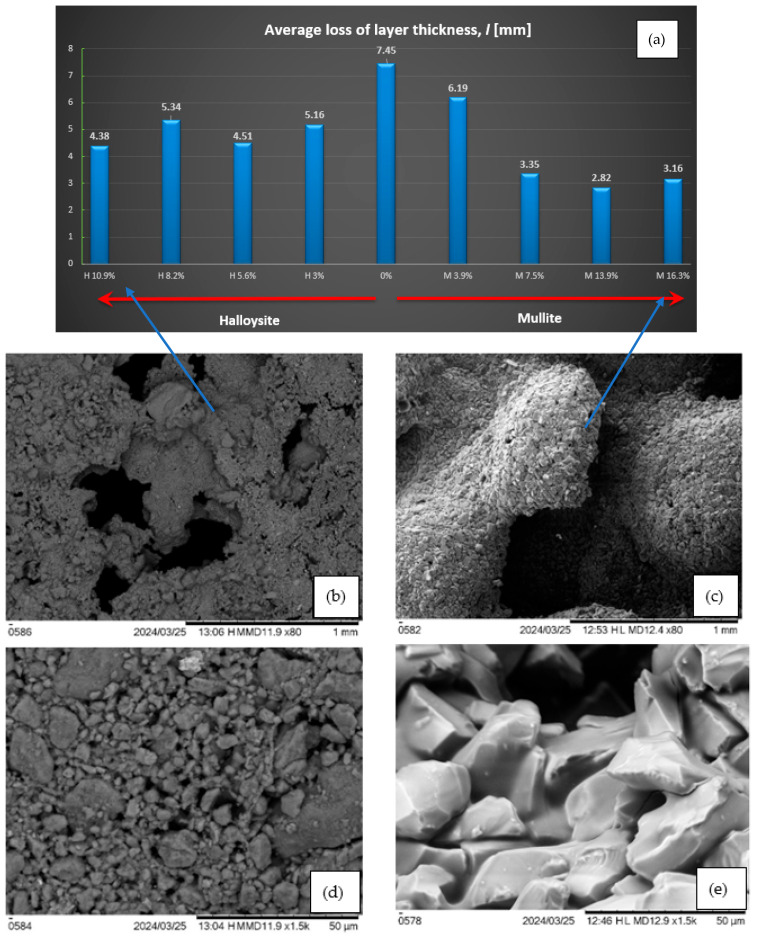
(**a**) Average loss of layer thickness l [mm]; SEM photo of the carbonized structure of the composite with the addition of (**b**) halloysite 10.9%, 80-fold approximation, (**c**) mullite 16.3%, 80-fold approximation, (**d**) halloysite 10.9%, 1500-fold approximation, and (**e**) mullite 16.3% zoom 1500 times.

The geometric reduction of the composite layer was also assessed. The values (see Figure 11a) represent the average thickness loss from each sample. In the case of mullite, there is a noticeable reduction in the thickness with the amount of additive (from 7.45 mm for composites without additive to only about 3 mm for 13.9% and 16.3% of mullite addition). However, for halloysite, with the addition of 10.9%, the thickness changes amounted to approximately 4 mm. There is a significant difference in the structure of the material after burning with the addition of mullite and halloysite (see Figure 11b–e).

## 4. Conclusions

Conducting ablation tests on the thermal protective properties of the powder composite with the addition of mullite and halloysite enabled the estimation of the temperature changes occurring on the back surface of the samples. A pyrometer was used to determine the approximate temperature of the front surface during the tests, while a thermal camera captured the temperature distribution on the back surface of the tested material. The use of double thermocouples facilitated a more accurate measurement of the rear surface temperature, addressing the uneven distribution confirmed by visual measurements from the thermal imaging camera. When feasible, positioning the thermocouples on the central surface allowed for precise readings of the desired temperature increases.

Based on this study, it can be concluded that the incorporation of powder reinforcement significantly enhanced the thermo-protective properties of the composite, particularly in terms of the mass ablation rate and ablative loss parameters, due to the increased proportion of the mullite additive. The addition of mullite and halloysite resulted in a notable reduction in the ablation parameters compared to the base material:

(1) Rear surface temperature from 58.9 °C to 49.2 °C (mullite) and to 49.8 °C (halloysite).
(2) Ablative weight loss from 57% to 18.9% (mullite) and up to 39.9% (halloysite).
(3) Speed of mass ablation from 77.9 mg/s to 25.2 mg/s (mullite) and up to 52.4 mg/s (halloysite).
(4) Loss of layer thickness from 7.4 mm to 2.8 mm (mullite) and up to 4.4 mm (halloysite).

The analysis of the obtained results from the experimental tests on ablative thermal protective properties proved a significant improvement in the thermal protective properties of the material. However, increasing the amount of mullite additive dynamically improved the ablation properties from just 7% of the additive amount, while adding halloysite improved the properties, but in a less dynamic way.

The obtained experimental results and their interpretation allow us to conclude that ablative coatings based on polymer composites with powder additives effectively influence thermal protection during short-term exposure to flammable gas streams. It is assumed that the use of a larger amount of the additive would result in an even greater reduction in temperature compared to the sample without the additive. Summarizing the obtained results, it should be noted that the impact of the use of the additive on the ablation properties was noted, but the differences are not very significant; therefore, in the future, additional tests should be carried out with a much higher share of the additive in the dispersion composite.

In the following steps, we plan to use computer vision to automatically and quantitatively analyze the surface char morphology, ablation front progression, and defect evolution from thermal and optical images. This will enable high-throughput, objective assessments beyond manual or single-point measurements. As future directions for our research, robust and efficient vision-based models could be employed, such as DeepLab [93]. DeepLabv3 is a deep neural network (DNN) architecture designed for semantic segmentation tasks. It uses atrous (dilated) convolutions to control receptive fields and feature map resolutions without increasing the total number of parameters. Another key feature is atrous spatial pyramid pooling, which effectively extracts multi-scale features that provide valuable information for segmentation. Overall, the network can generate dense feature maps with rich long-range information, allowing for accurate image segmentation. This technique involves labeling each pixel in an image with a class that corresponds to what that pixel represents. Another useful model is EfficientNet [94], which employs the idea of progressive learning. This means that, although training begins with small image sizes, the sizes increase gradually, addressing the slowdown in training speed that occurs with larger images.

## Figures and Tables

**Table 1:** Name, type of supplement and share wt. addition of samples.

No	Name	Type of Supplement	Share wt. Addition [%]
1.	1.1, 1.2, 1.3	Halloysite	10.9
2.	2.1, 2.2, 2.3		8.2
3.	3.1, 3.2, 3.3		5.6
4.	4.1, 4.2, 4.3		3.7
5.	5.1, 5.2, 5.3	0	0
6.	6.1, 6.2, 6.3	Mullite	3.9
7.	7.1, 7.2, 7.3		7.5
8.	8.1, 8.2, 8.3		13.9
9.	9.1, 9.2, 9.3		16.3

**Table 2:** Detailed data for each sample.

No.	Sample	Addition	Share	Mass Before Burning [g]	Mass After Burning [g]	Thickness Before Burning [mm]	Thickness After Burning [mm]	Loss of Layer Thickness [mm]	Ablative Loss of Mass [%]	Mass Speed of Ablation [mg/s]
**1**	1.1	halloysite	10.9	14.83	8.36	11.04	5.71	5.33	43.63	53.92
	1.2			15.55	9.87	11.76	7.47	4.29	36.53	47.33
	1.3			16.94	10.23	11.62	8.1	3.52	39.61	55.92
	average			–	–	–	–	4.38	39.92	52.39
**2**	2.1		8.2	16.32	9.5	12.24	6.44	5.8	41.79	56.83
	2.2			16.36	9.04	11.92	6.7	5.22	44.74	61
	2.3			15.2	8	11.58	6.57	5.01	47.37	60
	average			–	–	–	–	5.34	44.63	59.28
**3**	3.1		5.6	14.64	8.18	10.98	6.34	4.64	44.13	53.83
	3.2			15.37	8.66	11.35	7.62	3.73	43.66	55.92
	3.3			16.01	9.82	11.91	6.76	5.15	38.66	51.58
	average			–	–	–	–	4.51	42.15	53.78
**4**	4.1		3.0	16.15	9.83	12.11	7.13	4.98	39.13	52.67
	4.2			16.22	9.65	12.38	7.02	5.36	40.51	54.75
	4.3			16.58	9.63	12.08	6.95	5.13	41.92	57.92
	average			–	–	–	–	5.16	40.52	55.11
**5**	5.1	no addition	0	16.12	4.77	12.43	4.51	7.92	70.41	94.58
	5.2			16.44	7.72	12.72	5.21	7.51	53.04	72.67
	5.3			16.72	8.74	12.79	5.88	6.91	47.73	66.5
	average			–	–	–	–	7.45	57.06	77.92
**6**	6.1	mullite	3.9	14.39	4.03	10.28	3.32	6.96	71.99	86.33
	6.2			14.75	5.72	11.04	3.88	7.16	61.22	75.25
	6.3			14.65	6.19	11.07	6.61	4.46	57.75	70.5
	average							6.19	63.65	77.36
**7**	7.1		7.5	15.21	10.01	10.69	7.32	3.37	34.19	43.33
	7.2			15.28	11.12	11.36	7.63	3.73	27.23	34.67
	7.3			15	11.79	11.38	8.44	2.94	21.40	26.75
	average							3.35	27.60	34.92
**8**	8.1		13.9	16.97	13.24	11.61	8.58	3.03	21.98	31.08
	8.2			16.65	11.9	11.68	9.85	1.83	28.53	39.58
	8.3			16.56	12.48	11.66	8.07	3.59	24.64	34
	average							2.82	25.05	34.89
**9**	9.1		16.3	16.41	13.36	11.71	8.02	3.69	18.59	25.41
	9.2			16.21	13.71	11.65	9.14	2.51	15.42	20.83
	9.3			15.73	12.19	11.08	7.81	3.27	22.50	29.5
	average							3.16	18.84	25.25

## Data Availability

The original contributions presented in this study are included in the article. Further inquiries can be directed to the corresponding authors.
